# Diffusion tensor imaging studies on subjects with suicidal thoughts and behaviors: A descriptive literature review

**DOI:** 10.1002/brb3.2711

**Published:** 2022-08-09

**Authors:** Emanuele Zanghì, Francesco Corallo, Viviana Lo Buono

**Affiliations:** ^1^ IRCCS Centro Neurolesi “Bonino Pulejo” Messina Italy

**Keywords:** diffusion tensor imaging, suicidal behavior, white matter

## Abstract

**Objective:**

Globally, suicide represents the second leading cause of death in young people aged 15–29 years for both sexes, after traffic accidents. Suicide occurs not only in high‐income countries, in high‐income countries but it is a global phenomenon in all regions of the world and it represents a serious public health problem.

**Method:**

This review was conducted on studies focused on white matter alterations in people who have attempted or thought about suicide. We searched PubMed and Scopus databases and screened references of included studies and reviewed articles for additional citations. From the initial 21 publications, we included only 12 studies that met search criteria and described the association between white matter alterations and suicide.

**Results:**

White matter alterations in suicidal behaviors were found in the prefrontal cortex, orbitofrontal cortex, internal capsule, corpus callosum, and default mode networks, which are critical cerebral areas involved in emotion processing and regulation, decision‐making, executive functions, and empathy.

**Conclusions:**

White matter alteration in cerebral areas involving high cognitive process and emotional regulation to confer a heightened vulnerability for suicidal behavior. Suicide is a complex process ranging from suicidal ideation to planning, attempting, or committing suicide. The identification of abnormalities in underlying neural circuitry may help delineate the neurobiological basis for suicide risk.

## 1 INTRODUCTION

Around 800,000 people die by suicide every year, or one person every 40 s. Globally, suicide is the second leading cause of death in young people aged 15–29 years for both sexes, after traffic accidents . While the number of nonfatal suicidal behavior is 10−20 times higher (World Health Organization, 2019). Suicide is defined as death caused by self‐directed injurious behavior with the intent to die as a result of the behavior; a suicide attempt is defined as a nonfatal, self‐directed, potentially injurious behavior with the intent to die as a result of the behavior, even if the behavior does not result in injury; and suicidal ideation is defined as thinking about, considering, or planning suicide (Klonsky et al., 2016). Some studies showed White Matter (WM) alterations in individuals who have attempted suicide (Bessette et al., 2013; Jia et al., 2013; Kim et al., 2015).

More specifically, diffusion tensor imaging (DTI) studies on subjects with suicidal thoughts and behaviors (STBs) have reported lower fractional anisotropy (FA) in the default mode network (DMN), which includes the posterior cingulate cortex, dorsal anterior cingulate cortex, prefrontal cortex (Chase et al., 2017). The DMN is involved in multiple cognitive and affective functions, such as emotional processing, self‐referential mental activity, mind wandering, recollection of experiences, and likely exerts a modulatory role during attentional demanding tasks (Raichle, 2015). These data suggest that alterations in the emotion regulation network may contribute to suicidal behavior.

The predictive value of currently identified risk factors for suicide is limited, and a reliable biological risk marker has not yet been identified. Identification of WM alterations in thought and behavior suicidal could be instrumental in identifying prevention targets.

The descriptive review focused on the studies that investigated WM alterations in STBs and emotional and cognitive factors involved in suicide risk published to date and summarize the progress achieved in elucidating neurobiological substrates.

## 2 MATERIALS AND METHODS

A review was conducted on DTI studies investigating WM in people with STBs. Studies were identified by searching PubMed and Scopus databases from 2007, the year of the first‐related published article—on WM alterations and suicidal risk—to November 2020. Search keywords were “suicidal behavior white matter diffusion tensor imaging.” (suicidal[All Fields] AND (“behavior”[MeSH Terms] OR behavior[Text Word]) AND “white matter” [MeSH Terms] OR (“white” [All Fields] AND “matter” [All Fields] OR “white matter” [All Fields]) AND “diffusion tensor imaging” [MeSH Terms] OR “diffusion” [All Fields] AND “tensor” [All Fields] AND “imaging” [All Fields]) OR “diffusion tensor imaging” [All Fields]). We selected only English language studies and human subjects. Articles were selected based on title, abstract, and text. Studies were included after they fulfilled the following criteria:

1. Published peer‐reviewed research
2. Studies that specifically assessed the relationship between WM and cognitive functions
3. Studies using standardized neuropsychological measures
4. Study with a control group

We exclude:

1. Studies on subjects with neurological or major medical conditions that affect the brain
2. Case studies

## 3 RESULTS

**FIGURE 1:**
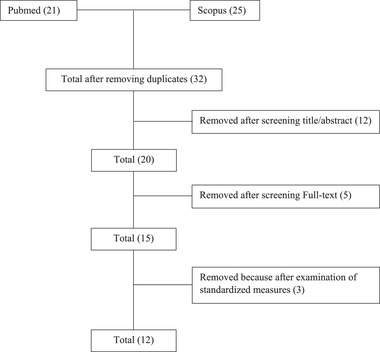
Search and selection of eligible articles

Of the 21 articles identified, only 12 articles met the inclusion criteria (Figure 1). All studies investigated the relationship between STBs and WM alterations in suicide attempts (SA) (see Table 1). All studies researched 375 subjects with suicidal ideation; 295 psychiatric subjects without suicidal ideation 444 control subjects. Subjects were within a range of ages between 18 and 65.

**TABLE 1:** Neuroimaging of STBs—key features of brain‐imaging studies

Study	Design	Aim of the study	Subjects	Results
Olvet et al. (2014)	DTI	Identify the altered WM regions in individuals with a prior SA	13 SAs (6 males, 7 females); 39 nonattempters (15 males, 24 females); 46 healthy participants (25 males, 21 females). Age range: 20 to 48 years old	Attempters had lower FA than nonattempters and HC in the dorsomedial PFC.
Chase et al (2017)	fMRI; DTI	Examine connectivity within the DMN within a group of young adults displaying high levels of suicidal ideation	34 suicidal ideators (7 males, 27 females; mean age: 24.41) and 40 healthy control participants (17 males, 23 females; mean age: 22.15)	Ideators has lower coupling between the dACC and dorsal PCC, compared to the dACC and ventral PCC.
Cyprien et al. (2016)	DTI	Investigate the specific impact of suicidal behavior on CC integrity in mood disorders	121 women (18 to 50‐year‐old): 41 with BD, 50 with MDD and 30 healthy controls	FA values for all CC regions were significantly lower in SAs. A higher number of SA were associated with significant splenium alterations.
Jia et al. (2010)	DTI	Use magnetic resonance DTI to characterize abnormalities of WM integrity in MDD patients with and without a history of SA	52 patients with MDD (mean age: 34.45), with (5 males, 11 females) and without (20 Male, 16 Female), history of SA, and 52 healthy controls (24 males, 28 females; mean age: 37.1)	FA was decreased in the left anterior limb of the internal capsule in SAs.
Jia et al. (2013)	DTI	Determine whether particular target fields of fibre projections through the ALIC are affected in MDD patients who recently attempted suicide	63 patients with MDD (age range: 19 to 50 years old): 23 with (8 Male, 15 Female), 40 without (21 Male, 19 Female)history of SA and 46 healthy controls (21 Male, 25 Female. Age range: 21 to 44 years old)	Those with a history of SA had greater abnormalities than those without SA in the left OFC and thalamus.
Johnston et al. (2017)	DTI; fMRI; MRI	Investigate implicated abnormalities in structural and functional connectivity within frontolimbic systems of adolescents/young adults with BD with and without history of SA	68 with BD (age range: 14 to 25 years old): 26 with suicide attempt (6 Male, 20 Female) 42 without attempts (19 Male, 23 Female). 45 healthy controls (age range: 15 to 25 years old. 26 Male, 19 Female)	Attempter group showed reductions in GM volume in OFC, hippocampus and cerebellum; WM integrity in UF, ventral frontal, and right cerebellum regions.
Myung et al. (2016)	DTI	Investigate whether or not MDD patients with suicidal ideation have different topological organizations of WM networks compared with MDD patients without suicidal ideation	24 patients with MDD and suicidal ideation (3 males, 21 females), 25 MDD patients without suicidal ideation (2 males, 23 females) and 31 healthy controls (9 males, 22 females; mean age: 55.5)	Reduced structural connectivity in a characterized subnetwork was found in patients with MDD and suicidal ideation.
Mahon et al. (2012)	DTI	Investigate WM integrity among BD patients with and without a history of SA compared to age‐ and sex‐matched healthy volunteers	14 BD patients with a SA (mean age: 33.3; 9 males, 5 females), 15 BD patients with no SA (mean age: 36.5, 9 males, 6 females), 15 healthy volunteers (mean age: 33.7; 8 males, 7 females)	BD patients with a prior SA had lower FA within the left orbital frontal WM.
Lischke et al. (2017)	DTI	Explore whether structural alterations of the CC are associated with emotional instability, impulsivity and suicidality in BPD	21 women with BPD (mean age: 26.21) and 20 healthy women control (mean age: 26.81).	Suicidal BPD participants showed less FA and more MD in these regions than HC participants but that nonsuicidal BPD participants.
Bijttebier et al (2015)	DTI; MRI	Identify structural network changes associated with a diathesis by examining the structural connectivity between regions in euthymic SAs	13 euthymic patients with a history of SA (4 males, 9 females; mean age: 31), 15 euthymic patients without a history of SA (7 males, 8 females; mean age: 36) and 17 healthy controls (1 male, 16 females; mean age: 26).	Decreased connectivity strength in SA in the connections between the left olfactory cortex and left anterior cingulate gyrus.
Zhang et al. (2019)	DTI	Explore the relationship between the CC, and BD and suicidal ideation	47 BD patients with suicidal ideation (16 males, 31 females), 59 with BD without suicidal ideation (26 males, 33 females), and 97 healthy controls (47 males, 50 females; age range: 15–50 years old)	BD patients with suicidal ideation had significant lower FA values than those of BD without suicidal ideation and HCs in the body and genu of the CC.
Kim et al. (2015)	DTI; MRI	Evaluate alterations in WM and GM in patients with PD with and without a history of SA	12 patients with PD and a history of SA (5 males, 7 females; mean age: 33.42) and 24 patients with PD and no history of SA (8 males, 16 females; mean age: 34)	Although no GM or WM volume differences were observed, increased FA values were found in the WM tracts of the SAs group compared with the non‐SAs group.

ALIC, anterior limb of the internal capsule; BD, bipolar disorder; BPD, borderline personality disorder; CC, corpus callosum; DMN, default mode network; dACC, dorsal anterior cingulate cortex; DTI, diffusion tensor imaging; FA, fractional anisotropy; fMRI, functional magnetic resonance imaging; GM, gray matter; HC, health control; MDD, major depressive disorder; MRI, magnetic resonance imaging; OFC, orbitofrontal cortex; PD, panic disorder; PCC, posterior cingulate cortex; SA, suicide attempt; SAs, suicide attempters; UF, uncinate fasciculus; WM, white matter.

For this review, we divided the different studies according to the involved cerebral areas:

1. Prefrontal cortex (PFC)
2. Orbitofrontal cortex (OFC)
3. Internal capsule
4. Corpus callosum (CC)

### 3.1 Prefrontal cortex

The prefrontal cortex (PFC) with its rich cortical and subcortical connections includes the ability to initiate and carry out new and goal‐directed patterns of behavior, sustained attention (Luria, 1966), filtering or gating mechanism for information processing (Shimamura et al., 1990), stimulus detection and sequencing tasks, planning (Lepage & Richer, 1996), encoding and retrieval of memory, scanning of In review 5 all the pertinent details (Siddiqui et al., 2008). Several studies showed the role of PFC in STBs. Gosnell et al. (2018) found reduced WM integrity in the medial PFC. Olvet et al. (2014) detected a lower FA in dorsomedial PFC. WM disruptions were found also in the frontal cortex–basal ganglia, areas with important implications for behavioral control (Cox Lippard et al., 2014). WM alterations were also found in frontolimbic networks, including uncinate fasciculus (UF). In particular, lower FA values in the main UF region seems to be associated with greater suicide ideation (Fan et al., 2019).

### 3.2 Orbitofrontal cortex

The orbitofrontal cortex (OFC) is involved in autonomic functions, response inhibition, and stimulus significance meaning (Damasio, 1996), mnemonic functions, and delayed response (Gold et al., 1996). This region has been shown to play a significant role in the anticipation and processing of outcomes even if the outcome does not produce any reward (Schnider et al., 2005) and in social and emotional behavior (Stuss & Benson, 1986).) Reduced WM integrity in the OFC was found in STBs compared to the control group (Gosnell et al., 2018). It has been hypothesized that WM disruptions in OFC impair decision‐making and emotional processing and may affect impulsivity in depressed and suicidal individuals. Another study showed that patients with a history of SA showed lower FA in the left OFC and greater impulsivity than patients without a history of suicide attempts (Johnston et al., 2017). In addition, lower FA in the OFC correlated significantly with motor impulsivity (Mahon et al., 2012).

### 3.3 Internal capsule

The internal capsule (IC) is a two‐way tract for the transmission of information to and from the cerebral cortex. Fiber tracts in the anterior limb are associated with emotion processing cognition, decision making, and motivation. WM abnormalities in the anterior limb have been found in psychiatric disease such as schizophrenia, bipolar disorder (Chowdhury et al., 2010) and obsessive‐compulsive disorder (Safadi et al., 2018). In suicidal patients, reduced FA has been observed in frontostriatal networks. This cerebral area, involved in executive planning and emotional regulation, affects suicidal behavior by disinhibiting emotional processes or interfering with rational judgment and planning (Gosnell et al. 2018). Other studies have found reduced FA in the right lentiform nucleus in SA compared to non‐SA (Kaschka & Rujescu, 2016). FA was decreased also in the left anterior limb of the internal capsule (Jia et al., 2010). Kim et al. (2015) found that aberrant WM integrity in the internal capsule and thalamic radiations was significantly associated with SA in patients with panic disorder compared with non‐SA patients.

### 3.4 Corpus callosum

The corpus callosum (CC) integrates and transfers information from both cerebral hemispheres to process sensory, motor, and high‐level cognitive signals (Goldstein et al., 2020; Tzourio‐Mazoyer, 2016). Cyprien et al. (2016) found a significantly lower FA in the splenium. Suicidal behavior has been associated with structural alterations in regions of the CC connected with prefrontal and temporoparietal brain regions implicated in emotion regulation, impulse control, and problem‐solving (Lischke et al., 2017). Zhang et al. (2019) reported that bipolar disorders (BD) patients with suicidal ideation presented a greater WM damage than BD patients without suicidal ideation.

## 4 DISCUSSION

Suicidal behavior is a multifaceted phenomenon affecting human populations, resulting from a complex interaction between genetic vulnerability, stress factors, underlying psychopathology and social aspects; however, the pathophysiological mechanism underlying this pathological behavior is still unclear (Lengvenyte et al., 2019). In recent years, neuroimaging studies have described micro and macrostructural abnormalities in WM on suicidal thoughts and behaviors subjects.

Suicidal behavior has been associated with functional abnormalities within the cingulate cortex (van Heeringen et al., 2014). In particular, a significant difference was found in the left inferior frontal gyrus between attempters and nonattempters. Other authors have described specific WM alteration in STBs in cerebral areas involved in the high cognitive processes, especially in PFC, OFC, IC, and CC (Andrews‐Hanna et al., 2010; Gosnell et al., 2018; Luria, 1966; Olvet et al., 2014). It has been theorized that suicidal behavior may be an attempt to escape the negative self‐view resulting from the perceived failure. Thus, we can hypothesize that alterations in WM PFC in SA may be related to self‐processing mismatch (Baumeister, 1990). Reduced FA SA has also been observed also in UF (Fan et al., 2019). Connections between the OFC and the amygdala, including reciprocal inhibitory connections, are critical for adaptive emotion regulation processes involved in mood disorders (Lippard et al., 2014; Olson et al., 2015). Lower FA in the UF region has been associated with higher most recent suicide ideation in the BD subjects. Other WM alterations have been found in OFC, which plays a significant role in social and emotional behavior (Stuss & Benson, 1986). These findings have been interpreted as a deficit in decision‐making and impulsivity related to SA (Gosnell et al., 2018). In particular, violent suicidal people generally make disadvantageous choices, i.e. they choose options with high immediate reward (Jollant et al., 2005), probably the alleviation of emotional pain, in the absence of the ability to generate future rewards (van Heeringen et al., 2011). In addition, SA showed reduced activation in the OFC for the contrast between disadvantageous and advantageous choices (Jollant et al., 2010). Other authors (Kaschka & Rujescu, 2016; Zhang et al., 2019) have reported in SA structural alterations in CC and deficits in emotion regulation and impulse control (Cyprien et al., 2011; Emsell et al., 2013; Gan et al., 2016; Matsuo et al., 2010)

It has been suggested that WM abnormalities also play a critical role in the pathogenesis of psychiatric disorders (Chen et al., 2021). DTI studies revealed disrupted connectivity within the DMN accompanying mood disorders (Hamilton et al., 2015) and rumination (Berman et al., 2011; Lois & Wessa, 2016). Moreover, decreased WM structural integrity, which provides frontolimbic connections in suicide attempters, has been found in both bipolar disorders and major depressive disorders (Fan et al., 2019). Other authors have suggested that individuals suffering from panic attacks symptoms are significantly associated with a lifetime history of SA (Katz et al., 2011; Nock et al., 2010) due to hyperarousal of the limbic system and activating of catastrophic cognitions that mutually amplify to produce and act on suicidal ideation (Katz et al., 2011). WM alterations in neural circuits involved in emotional processes and mood regulation can result in enhanced vulnerability to psychiatric morbidity (Serafini et al., 2015).

## 5 CONCLUSION

Microstructural alterations in WM could be considered a potential biomarker of suicidal ideation. The studies reviewed showed in SA WM changes in brain regions, such as PFC, CC and DMN, that are critical areas for emotion processing and regulation, decision‐making, executive functions and empathy.

A small number of papers were included in this review since only twelve studies met the inclusion criteria. In addition, due to the small sample size, the generalization of the clinical results is limited. Despite these limitations, this descriptive review underlines important implications for suicide behavior. To date, the possibility of identifying the underlying biological mechanisms to establish neuroanatomical correlates of suicide is very still limited. Further DTI studies on WM abnormalities could contribute to functional deficits and help to clarify the pathophysiological mechanisms underlying suicidal behavior in order to determine possible predictors of risk for future attempts and to develop more targeted and effective strategies for prevention.

## FUNDING

This research did not receive any specific grant from funding agencies in the public, commercial, or not‐for‐profit sectors. This study was supported by Current Research Funds 2022, Ministry of Health, Italy, provided to the IRCCS.

## CONFLICT OF INTEREST

The authors declare that there are no conflicts of interest.

### PEER REVIEW

The peer review history for this article is available at https://publons.com/publon/10.1002/brb3.2711

## Data Availability

The data that support the findings of this study are available on request from the corresponding author.

## References

[brb32711-bib-0001] Andrews‐Hanna, J. R. , Reidler, J. S. , Sepulcre, J. , Poulin, R. , & Buckner, R. L. (2010). Functional‐anatomic fractionation of the brain's default network. Neuron, 65, 550–562. 20188659 10.1016/j.neuron.2010.02.005 PMC2848443

[brb32711-bib-0002] Baumeister, R. F. (1990). Suicide as escape from self. Psychological Review, 97, 90–113. 2408091 10.1037/0033-295x.97.1.90

[brb32711-bib-0003] Berman, M. G. , Peltier, S. , Nee, D. E. , Kross, E. , Deldin, P. J. , & Jonides, J. (2011). Depression, rumination and the default network. Social Cognitive and Affective Neuroscience, 6, 548–555. 20855296 10.1093/scan/nsq080 PMC3190207

[brb32711-bib-0004] Bessette, K. L. , Nave, A. M. , Caprihan, A. , & Stevens, M. C. (2013). White matter abnormalities in adolescents with major depressive disorder. Brain Imaging and Behavior, 8(4), 531–541. 10.1007/s11682-013-9274-8 24242685

[brb32711-bib-0005] Bijttebier, S. , Caeyenberghs, K. , Van den Ameele, H. , Achten, E. , Rujescu, D. , Titeca, K. , & Van Heeringen, C. (2015). The vulnerability to suicidal behavior is associated with reduced connectivity strength. Frontiers in Human Neuroscience, 30(9), 632. 10.3389/fnhum.2015.00632 PMC4663245 26648857

[brb32711-bib-0006] Chase, H. W. , Segreti, A. M. , Keller, T. A. , Cherkassky, V. L. , Just, M. A. , Pan, L. A. , & Brent, D. A. (2017). Alterations of functional connectivity and intrinsic activity within the cingulate cortex of suicidal ideators. Journal of Affective Disorders, 1(212), 78–85. 10.1016/j.jad.2017.01.013 PMC5358995 28157550

[brb32711-bib-0007] Chen, V. C. H. , Kao, C. J. , Tsai, Y. H. , McIntyre, R. S. , & Weng, J. C. (2021). Mapping brain microstructure and network alterations in depressive patients with suicide attempts using generalized Q‐sampling MRI. Journal of Personalized Medicine, 11(3), 174. 33802354 10.3390/jpm11030174 PMC7998726

[brb32711-bib-0008] Chowdhury, F. , Haque, M. , Sarkar, M. , Ara, S. , & Islam, M. (2010). White fiber dissection of brain; the internal capsule: A cadaveric study. Turkish Neurosurgery, 20(3), 314–322. 20669103 10.5137/1019-5149.JTN.3052-10.2

[brb32711-bib-0009] Cox Lippard, E. T. , Johnston, J. A. , & Blumberg, H. P. (2014). Neurobiological risk factors for suicide: Insights from brain imaging. American Journal of Preventive Medicine, 47(3), Suppl 2 S152–S162. 25145733 10.1016/j.amepre.2014.06.009 PMC4143781

[brb32711-bib-0010] Cyprien, F. , Courtet, P. , Malafosse, A. , Maller, J. , Meslin, C. , Bonafé, A. , Le Bars, E. , de Champfleur, N. M. , Ritchie, K. , & Artero, S. (2011). Suicidal behavior is associated with reduced corpus callosum area. Biological Psychiatry, 70(4), 320–326. 21531383 10.1016/j.biopsych.2011.02.035

[brb32711-bib-0011] Cyprien, F. , de Champfleur, N. M. , Deverdun, J. , Olié, E. , Le Bars, E. , Bonafé, A. , Mura, T. , Jollant, F. , Courtet, P. , & Artero, S. (2016). Corpus callosum integrity is affected by mood disorders and also by the suicide attempt history: A diffusion tensor imaging study. Journal of affective disorders, 206, 115–124. 27472413 10.1016/j.jad.2016.07.026

[brb32711-bib-0012] Damasio, A. R. (1996). The somatic marker hypothesis and the possible functions of the prefrontal cortex. Philosophical Transactions of the Royal Society of London. Series B, Biological Sciences, 351,. 10.1098/rstb.1996.0125 8941953

[brb32711-bib-0013] Emsell, L. , Leemans, A. , Langan, C. , Van Hecke, W. , Barker, G. J. , McCarthy, P. , Jeurissen, B. , Sijbers, J. , Sunaert, S. , Cannon, D. M. , & McDonald, C. (2013). Limbic and callosal white matter changes in euthymic bipolar I disorder: An advanced diffusion magnetic resonance imaging tractography study. Biological Psychiatry, 73(2), 194–201. 23158457 10.1016/j.biopsych.2012.09.023

[brb32711-bib-0014] Fan, S. , Lippard, E. T. C. , Sankar, A. , Wallace, A. , Johnston, J. A. Y. , Wang, F. , Pittman, B. , Spencer, L. , Oquendo, M. A. , & Blumberg, H. P. (2019). Gray and white matter differences in adolescents and young adults with prior suicide attempts across bipolar and major depressive disorders. Journal of Affective Disorders, 245, 1089–1097. 30699851 10.1016/j.jad.2018.11.095 PMC6903411

[brb32711-bib-0015] Gan, J. , Yi, J. , Zhong, M. , Cao, X. , Jin, X. , Liu, W. , & Zhu, X. (2016). Abnormal white matter structural connectivity in treatment‐naive young adults with borderline personality disorder. Acta Psychiatrica Scandinavica, 134, 494–503. 27611589 10.1111/acps.12640

[brb32711-bib-0016] Gold, J. M. , Berman, K. F. , & Randolph, C. (1996). PET validation of a novel prefrontal task: Delayed response alternation. Neuropsychologia, 10, 3–10.

[brb32711-bib-0017] Goldstein, A. , Covington, B. P. , Mahabadi, N. , & Mesfin, F. B. (2020). Neuroanatomy, corpus callosum. StatPearls Publishing. 28846239

[brb32711-bib-0018] Gosnell, S. N. , Molfese, D. L. , & Salas, R. (2018). Brain morphometry: Suicide. Neuromethods, 136,.

[brb32711-bib-0019] Hamilton, J. P. , Farmer, M. , Fogelman, P. , & Gotlib, I. H. (2015). Depressive rumination, the default‐mode network, and the dark matter of clinical neuroscience. Biological Psychiatry, 78, 224–230. 25861700 10.1016/j.biopsych.2015.02.020 PMC4524294

[brb32711-bib-0020] Jia, Z. , Huang, X. , Wu, Q. , Zhang, T. , Lui, S. , Zhang, J. , Amatya, N. , Kuang, W. , Chan, R. C. , Kemp, G. J. , Mechelli, A. , & Gong, Q. (2010). High‐field magnetic resonance imaging of suicidality in patients with major depressive disorder. The American Journal of Psychiatry, 167, 1381–1390. 20843871 10.1176/appi.ajp.2010.09101513

[brb32711-bib-0021] Jia, Z. , Wang, Y. , Huang, X. , Kuang, W. , Wu, Q. , Lui, S. , Sweeney, J. A. , & Gong, Q. (2013). Impaired frontothalamic circuitry in suicidal patients with depression revealed by diffusion tensor imaging at 3.0T. Journal of Psychiatry & Neuroscience:JPN, 38, 130023. 10.1503/jpn.130023 PMC3997602 24119793

[brb32711-bib-0022] Johnston, J. A. Y. , Wang, F. , Liu, J. , Blond, B. N. , Wallace, A. , Liu, J. , Spencer, L. , Cox Lippard, E. T. , Purves, K. L. , Landeros‐Weisenberger, A. , Hermes, E. , Pittman, B. , Zhang, S. , King, R. , Martin, A. , Oquendo, M. A. , & Blumberg, H. P. (2017). Multimodal neuroimaging of frontolimbic structure and function associated with suicide attempts in adolescents and young adults with bipolar disorder. The American Journal of Psychiatry, 174(7), 667–675. 28135845 10.1176/appi.ajp.2016.15050652 PMC5939580

[brb32711-bib-0023] Jollant, F. , Bellivier, F. , Leboyer, M. , Astruc, B. , Torres, S. , Verdier, R. , Castelnau, D. , Malafosse, A. , & Courtet, P. (2005). Impaired decision making in suicide attempters. The American Journal of Psychiatry, 162, 304–310. 15677595 10.1176/appi.ajp.162.2.304

[brb32711-bib-0024] Jollant, F. , Lawrence, N. S. , Olie, E. , O'Daly, O. , Malafosse, A. , Courtet, P. , & Phillips, M. L. (2010). Decreased activation of lateral orbitofrontal cortex during risky choices under uncertainty is associated with disadvantageous decision‐making and suicidal behavior. Neuroimage, 51, 1275–1281. 20302946 10.1016/j.neuroimage.2010.03.027

[brb32711-bib-0025] Kaschka, W. P. , & Rujescu, D. (2016). Biological aspects of suicidal behavior. Psychoneuroendocrinology, 73(30), 110–122.

[brb32711-bib-0026] Katz, C. , Yaseen, Z. S. , Mojtabai, R. , Cohen, L. J. , & Galynker, I. I. (2011). Panic as an independent risk factor for suicide attempt in depressive illness: Findings from the National Epidemiological Survey on Alcohol and Related Conditions (NESARC). The Journal of Clinical Psychiatry, 72(12), 1628–1635. 21457675 10.4088/JCP.10m06186blu

[brb32711-bib-0027] Kim, B. , Oh, J. , Kim, M. K. , Lee, S. , Tae, W. S. , Kim, C. M. , Choi, T. K. , & Lee, S. H. (2015). White matter alterations are associated with suicide attempt in patients with panic disorder. Journal of Affective Disorders, 175, 139–146. 25617685 10.1016/j.jad.2015.01.001

[brb32711-bib-0028] Klonsky, E. D. , May, A. M. , & Saffer, B. Y. (2016). Suicide, Suicide Attempts, and Suicidal Ideation. Annual Review of Clinical Psychology, 12(1), 307–330. 10.1146/annurev-clinpsy-021815-093204 26772209

[brb32711-bib-0029] Lengvenyte, A. , Conejero, I. , Courtet, P. , & Olié, E. (2019). Biological bases of suicidal behaviours: A narrative review. The European Journal of Neuroscience, 53, 330–351. 31793103 10.1111/ejn.14635

[brb32711-bib-0030] Lepage, M. , & Richer, F. (1996). Inter‐response interference contributes to the sequencing deficit in frontal lobe lesions. Brain, 119, 1289–1295. 8813291 10.1093/brain/119.4.1289

[brb32711-bib-0031] Lischke, A. , Domin, M. , Freyberger, H. J. , Grabe, H. J. , Mentel, R. , Bernheim, D. , & Lotze, M. (2017). Structural alterations in the corpus callosum are associated with suicidal behavior in women with borderline personality disorder. Frontiers in Human Neuroscience, 24(11), 196. 10.3389/fnhum.2017.00196 PMC5401902 28484382

[brb32711-bib-0032] Lois, G. , & Wessa, M. (2016). Differential association of default mode network connectivity and rumination in healthy individuals and remitted MDD patients. Social Cognitive and Affective Neuroscience, 11, 1792–1801. 27405616 10.1093/scan/nsw085 PMC5091677

[brb32711-bib-0033] Luria, A. R. (1966). Higher cortical functions in man. Basic Books.

[brb32711-bib-0034] Mahon, K. , Burdick, K. E. , Wu, J. , Ardekani, B. A. , & Szeszko, P. R. (2012). Relationship between suicidality and impulsivity in bipolar I disorder: A diffusion tensor imaging study. Bipolar Disorders, 14(1), 80–89. 22329475 10.1111/j.1399-5618.2012.00984.x PMC3319758

[brb32711-bib-0035] Matsuo, K. , Nielsen, N. , Nicoletti, M. A. , Hatch, J. P. , Monkul, E. S. , Watanabe, Y. , Zunta‐Soares, G. B. , Nery, F. G. , & Soares, J. C. (2010). Anterior genu corpus callosum and impulsivity in suicidal patients with bipolar disorder. Neuroscience Letters, 469, 75–80. 19932153 10.1016/j.neulet.2009.11.047 PMC3412141

[brb32711-bib-0036] Myung, W. , Han, C. E. , Fava, M. , Mischoulon, D. , Papakostas, G. I. , Heo, J. Y. , Kim, K. W. , Kim, S. T. , Kim, D. J. , Kim, D. K. , Seo, S. W. , Seong, J. K. , & Jeon, H. J. (2016). Reduced frontal‐subcortical white matter connectivity in association with suicidal ideation in major depressive disorder. Translational Psychiatry, 6(6), e835. 27271861 10.1038/tp.2016.110 PMC4931608

[brb32711-bib-0037] Nock, M. K. , Hwang, I. , Sampson, N. A. , & Kessler, R. C. (2010). Mental disorders, comorbidity and suicidal behavior: Results from the National Comorbidity Survey Replication. Molecular psychiatry, 15(8), 868–876. 19337207 10.1038/mp.2009.29 PMC2889009

[brb32711-bib-0038] Olson, I. R. , Von Der Heide, R. J. , Alm, K. H. , & Vyas, G. (2015). Development of the uncinate fasciculus: Implications for theory and developmental disorders. Developmental Cognitive Neuroscience, 14, 50–61. 26143154 10.1016/j.dcn.2015.06.003 PMC4795006

[brb32711-bib-0039] Olvet, D. M. , Peruzzo, D. , Thapa‐Chhetry, B. , Sublette, M. E. , Sullivan, G. M. , Oquendo, M. A. , Mann, J. J. , & Parsey, R. V. (2014). A diffusion tensor imaging study of suicide attempters. Journal of Psychiatric Research, 51, 60–67. 24462041 10.1016/j.jpsychires.2014.01.002 PMC4060601

[brb32711-bib-0040] Raichle, M. E. (2015). The brain's default mode network. Annual Review of Neuroscience, 38, 433–447. 10.1146/annurev-neuro-071013-014030 25938726

[brb32711-bib-0041] Safadi, Z. , Grisot, G. , Jbabdi, S. , Behrens, T. E. , Heilbronner, S. R. , McLaughlin, N. C. R. , Mandeville, J. , Versace, A. , Phillips, M. L. , Lehman, J. F. , Yendiki, A. , & Haber, S. N. (2018). Functional segmentation of the anterior limb of the internal capsule: Linking white matter abnormalities to specific connections. The Journal of Neuroscience: The Official Journal of the Society for Neuroscience, 38(8), 2106–2117. 29358360 10.1523/JNEUROSCI.2335-17.2017 PMC5824744

[brb32711-bib-0042] Schnider, A. , Treyer, V. , & Buck, A. (2005). The human orbitofrontal cortex monitors outcomes even when no reward is at stake. Neuropsychologia, 43, 316–323. 15707609 10.1016/j.neuropsychologia.2004.07.003

[brb32711-bib-0043] Serafini, G. , Respino, M. , Murri, M. B. , & Amore, M. (2015). Diffusion tensor imaging techniques: Insights into alterations of white matter underlying major depression and suicidal behavior. Depression: A Silent Culprit in Health and Disease, 83,.

[brb32711-bib-0044] Shimamura, A. P. , Janowsky, J. S. , & Squire, L. R. (1990). Memory for the temporal order of events in patients with frontal lobe lesions and amnesic patients. Neuropsychologia, 28, 803–813. 2247207 10.1016/0028-3932(90)90004-8

[brb32711-bib-0045] Siddiqui, S. V. , Chatterjee, U. , Kumar, D. , Siddiqui, A. , & Goyal, N. (2008). Neuropsychology of prefrontal cortex. Indian Journal of Psychiatry, 50(3), 202–208. 19742233 10.4103/0019-5545.43634 PMC2738354

[brb32711-bib-0046] Stuss, D. T. , & Benson, D. F. (1986). The frontal lobes. Raven Press.

[brb32711-bib-0047] Tzourio‐Mazoyer, N. (2016). Intra‐ and inter‐hemispheric connectivity supporting hemispheric specialization. In: Kennedy, H. , Van Essen, D.C. , & Christen, Y. (Eds.). Micro‐, meso‐ and macro‐connectomics of the brain [Internet] (pp. 129–146). Springer. 28590670

[brb32711-bib-0048] van Heeringen, K. , Bijttebier, S. , Desmyter, S. , Vervaet, M. , & Baeken, C. (2014). Is there a neuroanatomical basis of the vulnerability to suicidal behavior? A coordinate‐based meta‐analysis of structural and functional MRI studies. Frontiers in Human Neuroscience, 8, 824. 25374525 10.3389/fnhum.2014.00824 PMC4205829

[brb32711-bib-0049] van Heeringen, C. , Bijttebier, S. , & Godfrin, K. (2011). Suicidal brains: A review of functional and structural brain studies in association with suicidal behaviour. Neuroscience & Biobehavioral Reviews, 35(3), 688–698. 20826179 10.1016/j.neubiorev.2010.08.007

[brb32711-bib-0050] World Health Organization . (2019). Suicide in the world: Global Health Estimates. Geneva, Switzerland: World Health Organization.

[brb32711-bib-0051] Zhang, R. , Jiang, X. , Chang, M. , Wei, S. , Tang, Y. , & Wang, F. (2019). White matter abnormalities of corpus callosum in patients with bipolar disorder and suicidal ideation. Annals of General Psychiatry, 18, 20. 31528196 10.1186/s12991-019-0243-5 PMC6737682

